# Stromal Neutrophil Extracellular Trap Density Is an Independent Prognostic Factor for Cervical Cancer Recurrence

**DOI:** 10.3389/fonc.2021.659445

**Published:** 2021-08-11

**Authors:** Bin Yan, Xuan Dai, Quanfu Ma, Xufeng Wu

**Affiliations:** Department of Gynecologic Oncology, Maternal and Child Health Hospital of Hubei Province, Huazhong University of Science and Technology, Wuhan, China

**Keywords:** polymorphonuclear neutrophils (PMNs), neutrophil extracellular traps (NETs), cervical cancer, recurrence-free survival (RFS), prognosis

## Abstract

**Background:**

Emerging evidence indicates that the tumor microenvironment influences tumor progression and patient prognosis through various inflammatory cells. Polymorphonuclear neutrophils (PMNs) and their functional structures termed neutrophil extracellular traps (NETs) are prominent constituents of several malignant tumors and affect the tumor microenvironment and cancer evolution. Here, we investigate the prognostic value of PMNs and NETs for recurrence in patients with cervical cancer.

**Methods:**

The study comprised 126 cervical cancer patients who were retrospectively enrolled. CD66b^+^ neutrophils and myeloperoxidase/citrullinated histone H3 (MPO/H3Cit)-labeled NETs were assessed by immunofluorescence, and the relationships with clinical and histopathologic features and patient outcome were evaluated.

**Results:**

The highest density of CD66b^+^ neutrophils were observed in the stromal compartment (median 55 cells/mm^2^). Above median densities of stromal CD66b^+^ neutrophils and NETs were significantly associated with short recurrence-free survival (RFS) (*P* = 0.041 and *P* = 0.006, respectively). Multivariate analysis identified high clinical stage (hazard ratio [HR] 6.40; 95% confidence interval [CI] 3.51-11.64; *P <* 0.001), lymph node metastases (HR 4.69; 95% CI 3.09-9.66; *P* = 0.006) and high density of NETs (HR 2.66; 95% CI 1.21-5.82; *P* = 0.015) as independent prognostic factors for short RFS, whereas a high density of CD66b^+^ neutrophils was not significant. Patients with a high NET density showed worse recurrence status in every stage, but the difference was only significant for stage I (*P* = 0.042), not stages II, III, or IV (all *P* > 0.05). Combining stromal NET density and the tumor, nodes, metastasis (TNM) staging system had better prognostic accuracy for cervical cancer than the TNM staging system alone at five and six years respectively (*P* = 0.010 and *P* = 0.023).

**Conclusion:**

Stromal NET density is an independent prognostic factor for RFS in cervical cancer. Combining NETs with the TNM staging system may further improve prognostic stratification.

## Introduction

Cervical cancer is the third most frequent cancer in the world and causes more than 270,000 deaths annually (1). Although advances in surgery, chemoradiotherapy, and immunotherapy have substantially improved outcomes, ∼40% of patients will experience recurrence after curative intent treatment and eventually die (2). The Union for International Cancer Control (UICC) tumor, nodes, metastasis (TNM) and the Federation International of Gynecology and Obstetrics (FIGO) classification system are used for prognostication and treatment recommendations in cervical cancer based on the anatomical extent of tumor, and the clinical outcome varies significantly among patients with the same tumor stage (3).

This “tumor cell-oriented” paradigm was recently replaced by a holistic vision including the tumor environment as a major player in cancer formation and development. Tumor behavior should now be considered as a balance between the tumor process and host reaction (4). Tumor progression is the product of evolving crosstalk between malignant cells and various stromal and immune cell subsets of the surrounding microenvironment (5). Immunological data (i.e., the type, density, and location of immune cells within the tumor samples) were found to be a better predictor of patient survival than histopathological methods currently used to stage a variety cancer such as colorectal, breast, and lung cancers (6, 7). However, in cervical cancer there are no prognostic factors that reflect the functional status in tumor environment and patients’ outcomes.

The tumor microenvironment represents an integral part of cancer (8) and is composed of cancer cells and various nonmalignant host cells (8, 9). Neutrophils (polymorphonuclear neutrophils, PMNs) comprise 50% to 75% of circulating leukocytes, constitute a significant portion of the tumor microenvironment, play major roles linking inflammation and cancer, and are actively involved in progression and metastasis, depending on their pro-tumoral or anti-tumoral status (10). Following stimulation, neutrophils can form structures called neutrophil extracellular traps (NETs) (11). These neutrophil-derived extracellular structures exist in the inflammatory environment and consist of decondensed DNA complexed with citrullinated histones (H3Cit) and various neutrophil granule proteins such as myeloperoxidase (MPO) and neutrophil elastase (NE) (12–14). They are usually generated in response to infectious stimuli and were initially described as a mechanism in the bactericidal effect and autoimmune diseases (15). Recent evidence has also linked NETs to a variety of cancers; they participate in tumor progression and are closely related to tumor proliferation, metastasis, and thrombosis (16, 17).

However, the correlation between NETs and cervical cancer remains almost unknown. A previous study reported that Peripheral blood neutrophils generate NETs in a subset of cervical cancer patients, while no NET formation is observed in peripheral blood neutrophils from healthy donors (18). NETs may also exist in the cervical cancer microenvironment.

Tissue microarrays (TMAs) are widely used in clinical and basic-science research, it is a powerful tool for undertaking large-scale tissue-based biomarker studies (19).The measurement of *in situ* NETs in most studies using hematoxylin and eosin (H&E)-based pathologist evaluation or single-color immunohistochemical technology is semi-quantitative and subjective, making it difficult to compare across laboratories (20). Flow cytometry, which fails to gain architectural information is similarly limited. Considering these disadvantages, we use multiplexed quantitative immunofluorescence for compartment-specific and *in situ* measurement of NETs in the tumor microenvironment, which can provide more sensitive, objective and superior prognostic information (21).

Hence, this clinical study sought to demonstrate whether NETs may serve as biomarkers in cervical cancer patients by using multiplexed quantitative immunofluorescence technology. Specifically, our aims were as follows: (i) to examine the formation of NETs in the tumor nests and stromal of cervical cancer tissues; (ii) to assess the correlations between the density of NETs in tumor nests and stromal of the tumor tissues and clinicopathologic features in cervical patients; (iii) to evaluate the correlations between the density of NETs in tumor nests and stromal of the tumor tissues and recurrence-free survival (RFS) as patient outcomes; (iv) to established a predictive model for cervical cancer by combining the TNM staging system and stromal NET density.

## Materials and Methods

### Patient Samples and Data

The human cervical cancer tissues microarray was purchased from Shanghai Outdo Biotech Company (HUteS169Su01, Shanghai, China) and included 126 paired cervical cancer tissues. Patients underwent surgery from January 2010 to October 2011, and follow-up information was available from February 2010 to March 2017. The study protocols were conducted in accordance with the principles expressed in the Declaration of Helsinki. Written informed consent was obtained from all patients before recruitment, and the study was conducted under the approval of the Institutional Ethics Committee.

Prior to entry, patients received a standard evaluation, including physical examination, cytological examination, colposcopy, biopsy, laboratory examinations and image examinations. The tumor samples were obtained by biopsy prior to any treatment. Two senior oncological gynecologists participated to evaluate patients’ clinical stage according to the Union for International Cancer Control (UICC) criteria 7th Edition and World Health Organization classification (22). The lymph node metastasis was defined by MRI of significantly enlarged lymph node (23). Inclusion criteria: (1) patients were pathologically diagnosed and had primary and previously untreated cervical cancer; (2) age younger than 75; (3) patients completed whole treatment and have complete follow-up data; (4) Eastern Cooperative Oncology Group (ECOG) performance status 0-2; (5) normal liver and renal function; and (6) no existing complicating disease or prior malignant disease. Treatment information was available as receipt of surgery, chemotherapy, or radiotherapy according to their clinical staging, but the intent of each treatment type could not be identified.

All tissue samples were collected from patients with cervical cancer who had not received any treatment. Standard biosecurity and institutional safety procedures have been adhered to. The clinicopathological characteristics of 126 subjects, including age, clinical stage, histology, lymph node metastasis, and TNM clinical stages are summarized in **Table 1**. Recurrence-free survival (RFS) was defined as the interval from the initial diagnosis to the date of disease progression or the date of the most recent follow-up.

**Table 1:** Clinicopathological characteristics of 126 patients at initial presentation.

Characteristics	No.	%
**Age**		
Median (range)	48 (29-70)	
**Clinical stage**		
I	69	54.8
II	30	23.8
III	24	19.0
Iv	3	2.4
**Histology**		
SCC	116	92.1
A	3	2.4
AS	6	4.8
Others	1	0.8
**Grade**		
1	1	0.8
2	13	10.3
3	91	72.2
unknown	21	16.7
**Lymph node**		
Negative	101	80.2
Positive	25	19.8
**Status**		
Survival	90	71.4
Death	36	28.6
**Recurrence**		
No	84	66.7
\Yes	42	33.3

A, adenocarcinoma; AS, adenosquamous carcinoma; SCC, squamous cell carcinoma.

### Multiplexed Immunofluorescence (MIF) and Quantification

TMA slides were stained using Opal 7-Color Manual IHC Kit (PerkinElmer, NEL811001KT, Waltham, MA, USA). Human appendicitis samples were used as a positive control, and human appendicitis slides were incubated with isotype control antibodies as a negative control. Briefly, the TMA slide was heated for 4 h in a dry oven at 60°C, deparaffinized (xylene 15 min two times, 100% ethanol, 95% ethanol, 85% ethanol, and 75% ethanol for 10 min), and rinsed in water for 10 min at room temperature (RT). After that, slide was pre-treated in Tris-EDTA buffer (pH 9.0) by microwaving treatment (MWT) (2 min on 100% power, followed by 15 min on 20% power). Then the slide was incubated with primary antibodies against CD66b, MPO, histone 3 (H3Cit), cytokeratin (CK), and 4’,6-diamidino-2-phenylindole (DAPI).

Primary antibody staining comprised three steps. First, tissue sections were covered with blocking buffer and incubated in a humidified chamber for 30 min at RT. Next, they were incubated with CK primary antibody (Kit-0009, MXBiotechnology, Fuzhou, China, dilution 1:50) in a humidified chamber at 4°C overnight followed by detection using Opal Polymer horseradish peroxidase mouse+rabbit for 20 min at RT. Visualization of CK was accomplished using Opal Fluorophore (dilution 1:100), after which the slide was placed in AR6 buffer and subjected to MWT (45 s at 100% power and additional 2 min at 50% power). In a serial fashion, the slide was then incubated with primary antibodies for CD66b (ARG65820, Arigobio, Taiwan, ROC, dilution 1:500), MPO (66177-1-Ig, Proteintech, Rosemont, IL, USA, dilution 1:1000), H3Cit (Ab5103, Abcam Inc, Cambridge, UK, dilution 1:3000) repeating the above steps using different Opal fluorophores (CK, Opal 520; CD66b, Opal 570; H3Cit, Opal 620; MPO, Opal 650). Finally, nuclei were visualized with DAPI working Solution for 5 min at RT in a humidity chamber, and the section was cover slipped with mounting medium (0100-01, SouthernBiotech, Birmingham, AL, USA).

The detailed information for primary antibodies and the correspondent Opal fluorophores is summarized in **Supplementary Table S1**.

### Automated Image Acquisition and Quantification

Briefly, whole tumor section images were captured with a Vectra Imaging System (version 2.0.8, PerkinElmer) with a 20× objective lens under the same bit depth, laser power, and exposure time to ensure comparability. InForm 2.1.1 image analysis software (PerkinElmer) was used to perform batch analysis of these Vectra-created image files as previously described (24). Each cell was identified by detecting the nuclear spectral element (DAPI). The CK-positive region was defined as the tumor nest region. The stromal region excluded the tumor nest region from the DAPI area. Neutrophils were identified as CD66b-positive cells in the tissue. NETs were identified as MPO/H3Cit-positive cells in the tissue (25). PMNs and NETs in different regions were quantified by dividing the densities of PMNs and NETs in the compartment by the area of the corresponding region, and the data are expressed as positive cells per million pixels (26).

PMN and NETs were recorded as positive when their optical density was above the signal detection threshold (6.078205 for MPO, 22.24 for H3Cit and 7.84 for CD66b), which was determined by the negative controls and visual inspection. The total number for each sample was used for final analysis. Images with <3% tumor tissue or staining artifacts were excluded from the analysis.

### Statistics

All sample data are expressed as numerical values or percentages. Chi-square tests were used to compare the baseline characteristics of the basic clinical information of patients (27). Survival curves were generated and evaluated using the Kaplan–Meier method for prognostic factors. Survival differences were compared using log-rank tests (28, 29). Cox regression models were built to analyze risk factors for survival outcomes in cervical cancer patients. Multivariate analyses with a Cox proportional hazards model were used to test independence, significance, and hazard discrimination (30). Time-dependent receiver operating characteristic (ROC) curve analysis was performed to determine the efficacy of TNM staging combined with density of PMNs and NETs for survival prognosis (31). Statistical analyses were performed using SPSS Statistics, Version 21.0 (IBM, Corp) and R software, version 4.0.2. Packages for “survival” and “timeROC” were used (http://www.r-project.org/). *P* value < 0.05 was considered statistically significant.

## Results

### Patient Characteristics

Patient characteristics are listed in **Table 1**. A total of 126 patients with clinically and histologically confirmed cervical cancer were included. The mean age at diagnosis was 48 years (range, 29–70 years); 69 (54.8%) were stage I, 30 (23.8%) were stage II, 24 (19%) were stage III, and the remaining 3 (2.4%) were stage IV. Pathologic analysis revealed that 116 (92.1%) of the patients had squamous cell carcinoma, and 25 (19.8%) of patients were lymph node positive. By the last follow-up, 36 (28.6%) of the patients had died. The estimated 5-year RFS rate was 71% (95% CI, 67% to 76%), and the median time to death was 64 months (range, 8 to 86 months).

### Tumor-Infiltrating Neutrophils Correlated With RFS

**Figure 1:**
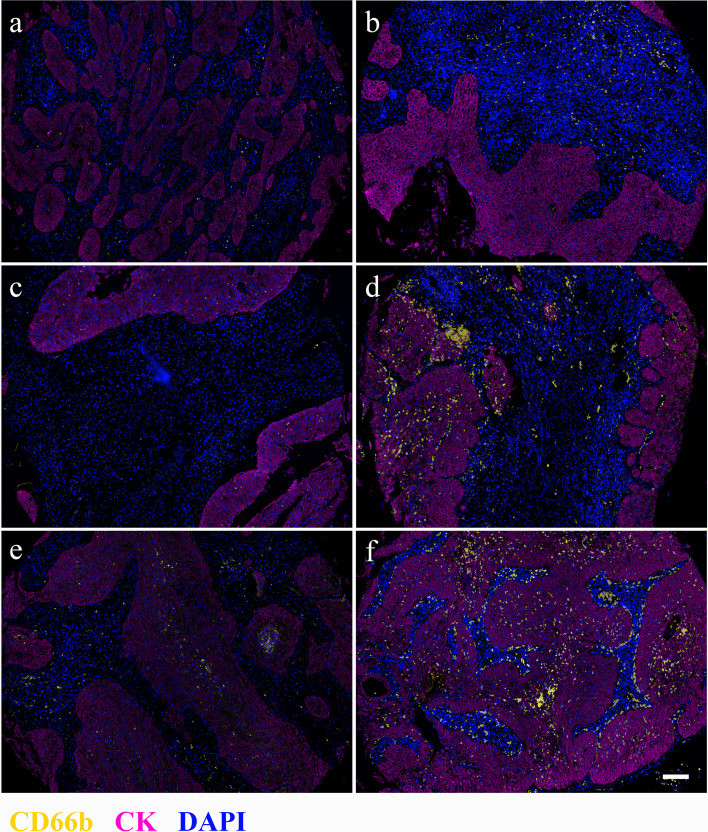
Distribution of CD66b^+^ cells in stromal **(A, B)**, tumor nest **(C, D)**, and whole tissues of cervical cancer samples. Left **(A, C, E)**, low CD66b density; right **(B, D, F)**, high CD66b density. Magnification 4×, scale bar = 100 μm. CD66b, yellow; CK, magenta; DAPI, blue.

Neutrophils were detected by IF staining of CD66b in paraffin-embedded tissues from 126 untreated patients with cervical cancer. Infiltration (distinguished by CK staining) was classified as located in tumor nests or stromal tissue as shown in **Figure 1**. The median densities of neutrophils in the stromal tissue, tumor nests, and whole samples were 55 cells/mm^2^ (range: 0–311), 13 cells/mm^2^ (range: 0–115) and 67 cells/mm^2^ (range: 0–354), respectively.

The highest mortality rate was in patients with the highest density (quartile 4) of neutrophils in the total tissue and stroma compartments, but not in tumor nests (**Table 2**). The 5-year RFS was 64% for patients with stromal neutrophils in quartile 4 compared with 86% for patients with stromal neutrophils in quartile 1.

**Table 2:** Percentage of patients with recurrence within 5 years from diagnosis stratified by CD66b^+^ neutrophil cell density quartiles.

Quartiles	Cells per mm^2^	% patients with recurrence
Total neutrophils		
I	0-15	22
II	15.1-47	20
III	47.1-90	22
IV	90.1-354	36
Stromal neutrophils		
I	0-13	14
II	13.1-40	22
III	40.1-65	28
IV	65.1-311	36
Tumor nest neutrophils		
I	0-1	25
II	1.1-4	33
III	4.1-16.25	20
IV	16.26-115	22

**Figure 2:**
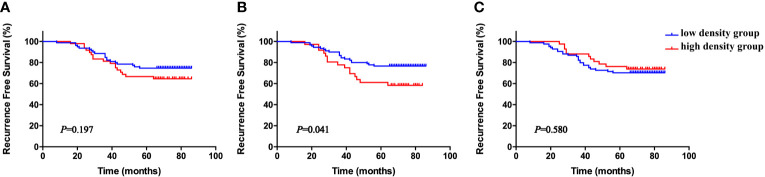
Kaplan–Meier RFS curves according to density of total tissue CD66b^+^ neutrophils **(A)**, stromal CD66b^+^ neutrophils **(B)**, and tumor nest CD66b^+^ neutrophils **(C)**, all stratified by medians. *P*-values obtained from log-rank tests. RFS, recurrence-free survival.

The cohort was divided into six groups according to the median neutrophil densities in the whole tumor tissues, stromal tissues, and tumor nests. As shown in **Figure 2**, high CD66b^+^ neutrophil density in stromal tissue was significantly associated with short RFS (*P* = 0.041), whereas the densities of neutrophils in the whole sample and within the tumor nests were not (*P* = 0.197, *P* = 0.580, respectively). The correlations between stromal CD66b^+^ cell density and clinicopathological parameters are detailed in **Table 3**. The high density of stromal CD66b^+^ cells was significantly associated with clinical stage (*P* = 0.006). There was no association between stromal CD66b^+^ cells and histology, pathologic grade, or lymph node involvement. This suggests that high neutrophil density in stromal tumor tissue is positively correlated with cervical cancer progression. There was no statistical difference between patients with high and low densities of stromal CD66b^+^ cells in different stages (data not shown).

**Table 3:** Baseline characteristics of patients with low- and high-density stromal PMNs.

	Low density (n = 90)		High density (n = 36)		*P*
	No.	%	No.	%	
Median age (range)	48 (29-70)		47 (30-68)		0.741
Clinical stage					0.007
I/II	74	82.2	25	69.4	
III/IV	16	17.8	11	30.6	
Histology					0.447
SCC	84	93.4	32	88.9	
No SCC	6	6.6	4	11.1	
Grade					0.238
1-2	10	11.1	4	11.1	
3	60	66.7	31	86.1	
Unknown	20	22.2	1	2.8	
Lymph node					0.056
Negative	76	84.4	25	69.4	
Positive	14	15.6	11	30.6	

PMN, polymorphonuclear neutrophil; SCC, squamous cell carcinoma.

### NET Accumulation Correlated With Shorter Survival

**Figure 3:**
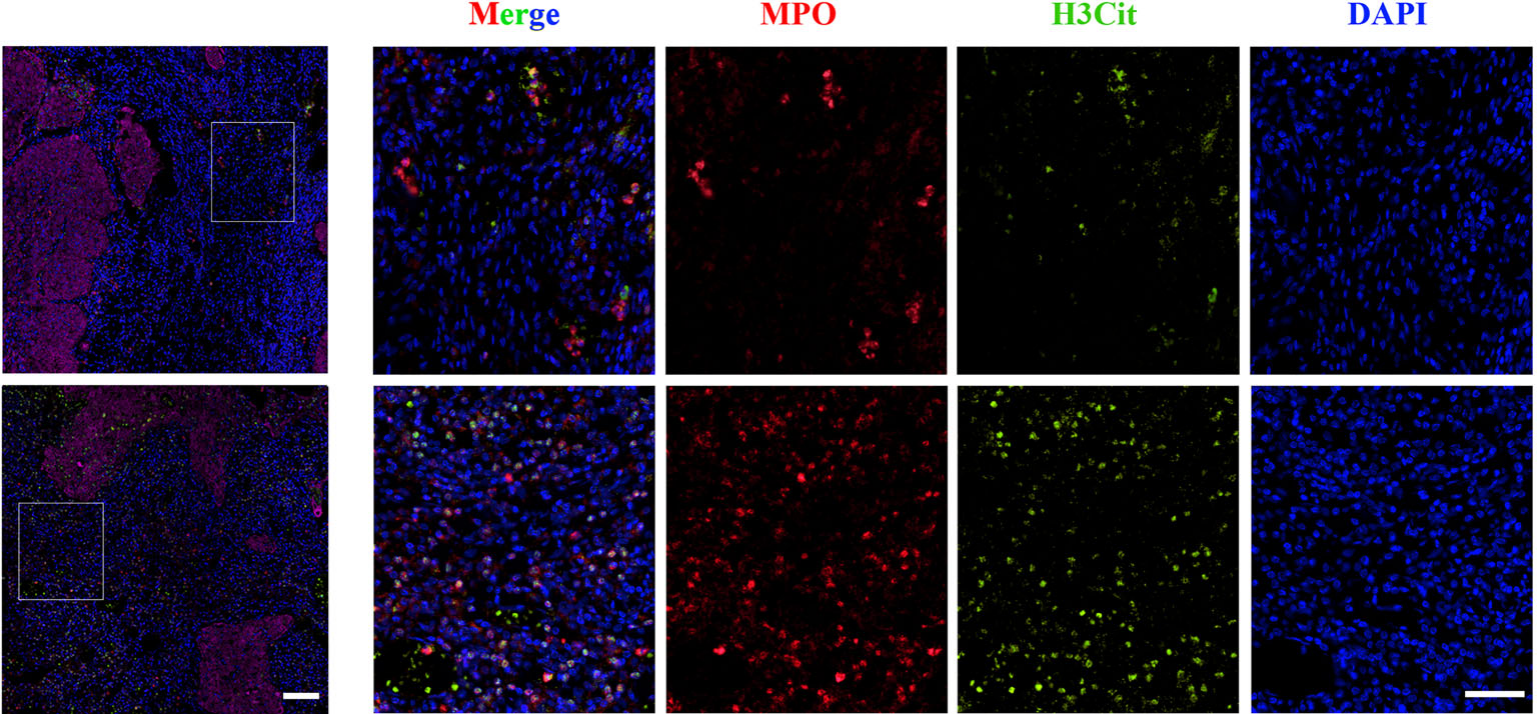
Representative images of immunofluorescence staining of low (upper panel) and high (lower panel) NET density in cervical cancer samples. Magnification 4×, scale bar = 100 μm; magnification 20×, scale bar = 100 μm. MPO, Red; H3Cit, Green; DAPI, Blue.

NETs were detected in the tissue samples mentioned above with double IF for MPO and H3Cit. They were classified according to tumor compartment (distinguished by CK staining) in tumor nests and stromal tissue as shown in **Figure 3**. The median densities of NETs in the whole tissue, stromal tissue, and tumor nests were 9 cells/mm^2^ (range 0-79), 5 cells/mm^2^ (range 0-32) and 4 cells/mm^2^ (range 0-51), respectively.

The highest rate of death was noted for patients with the highest density (quartile 4) of NETs in stromal compartments, but not in total tissue or tumor nests (**Table 4**). The 5-year RFS was 67% for patients with stromal NET density in quartile 4 compared with 86% for patients with stromal NET density in quartile 1.

**Table 4:** Mortality within 5 years of cervical cancer diagnosis stratified by NET density quartile.

Quartiles	Cells per mm^2^	% patients with recurrence
Total NETs		
I	0	11
II	0.1-5	31
III	5.1-13	47
IV	13.1-79	11
Stromal NETs		
I	0	14
II	0.1-2	22
III	2.1-5.25	31
IV	5.26-32	33
Tumor nest NETs		
I	0	36
II	0.1-2	33
III	2.1-425	11
IV	4.26-51	20

**Figure 4:**
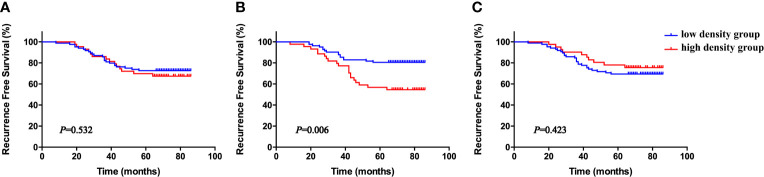
Kaplan–Meier RFS curves according to density of whole tissue NETs **(A)**, stromal NETs **(B)**, and tumor nest NETs **(C)**, all stratified by medians. *P*-values obtained from log-rank tests. RFS, recurrence-free survival.

Again, the patients were divided into six groups according to the median NETs density in the whole tumor tissues, stromal tissues, and tumor nests. As shown in **Figure 4**, high stromal tissue NET density was significantly associated with short RFS (*P* = 0.006), whereas the densities of NETs in the whole tissue sample and within tumor nests were not (*P* = 0.532, *P* = 0.423, respectively). The correlations between stromal NET density and clinicopathological parameters are given in **Table 5**. There was no association between stromal NET density and stage, histology, pathologic grade, or lymph node involvement. This result suggests that high NET density in tumor tissue stromal is inversely correlated with cervical cancer prognosis.

**Table 5:** Baseline characteristics of patients with cervical cancer in low- and high-density groups of stromal PMNs.

	Low density (n = 81)		High density (n = 45)		*P*
	No.	%	No.	%	
Median age (range)	47 (29-70)		48 (30-70)		0.397
Clinical stage					
I/II	67	82.7	32	71.1	0.393
III/IV	14	17.3	13	28.9	
Histology					
SCC	74	91.4	42	93.3	0.369
No SCC	7	8.6	3	6.7	
Grade					
1-2	7	8.6	7	15.6	0.348
3	58	71.6	33	73.3	
Unknown	16	19.8	5	11.1	
Lymph node					
Negative	69	85.2	32	71.1	0.058
Positive	12	14.8	13	28.9	

PMN, polymorphonuclear neutrophil; SCC, squamous cell carcinoma.

### Multivariate Analysis

A multivariate Cox proportional-hazard regression model was used to analyze the relative strength and potential independence of CD66b^+^ neutrophil and NET density. For comparability, estimates from the stromal compartment were chosen for the model. Univariate analysis was carried out to identify clinical characteristics that were significantly associated with RFS (**Table 6**), such as clinical stage, histology, grade, lymph node metastasis, and stromal PMN and NET densities. Clinical stage, lymph node metastasis, and stromal PMN and NET densities were found to have significant effects on RFS and were included in the subsequent multivariate analysis. The results revealed that clinical stage, stromal NET density, and lymph node metastasis were significant independent predictors of poor RFS (**Table 3**), but a high density of stromal CD66b^+^ neutrophils was not significant.

**Table 6:** Univariate and multivariate analyses of the association of different variables with RFS.

Variable	Univariate		Multivariate	
	HR (95% CI)	*P*	HR (95% CI)	*P*
Clinical stage	3.413 (2.307-5.050)	<0.001	6.396 (3.514 -11.641)	<0.001
Histology	1.207 (0.655-2.222)	0.547		
Grade	1.335 (0.500-3.566)	0.564		
Lymph node	4.487 (2.316-8.693)	<0.001	4.689 (3.090-9.663)	0.006
PMN stromal density	1.964 (1.012-3.811)	0.046	0.793 (0.371-1.695)	0.550
NET stromal density	2.523 (1.307-4.873)	0.006	2.655 (1.212-5.818)	0.015

CI, confidence interval; HR, hazard ratio; NET, neutrophil extracellular trap; PMN, polymorphonuclear neutrophil; RFS, recurrence-free survival.

**Figure 5:**
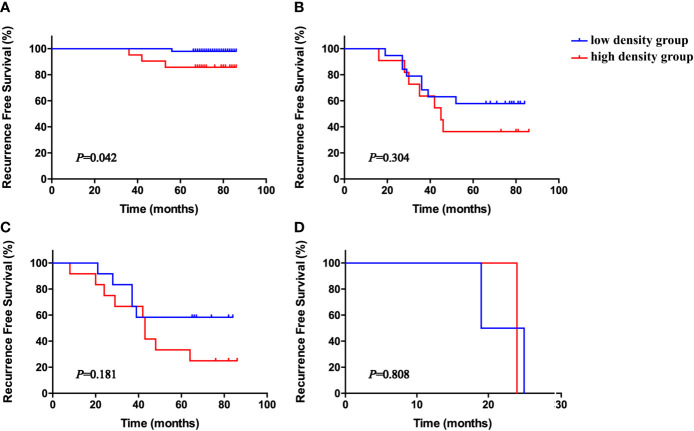
Kaplan–Meier RFS curves according to stromal NET density in stage I **(A)**, II **(B)**, III **(C)**, and IV **(D)**, all stratified by medians. *P*-values obtained from log-rank tests. RFS, recurrence-free survival.

To further evaluate the prognostic role of stromal NET density in different subgroups, patients were stratified according to TNM stage. High stromal NET density was associated with worse survival status in stages I, II, and III, but the results were only significant for stage I (**Figure 5**, *P* = 0.042). There were no significant differences for stages II, III, and IV (**Figure 5**, *P* = 0.304, *P* = 0.181 and *P* = 0.808, respectively).

**Figure 6:**
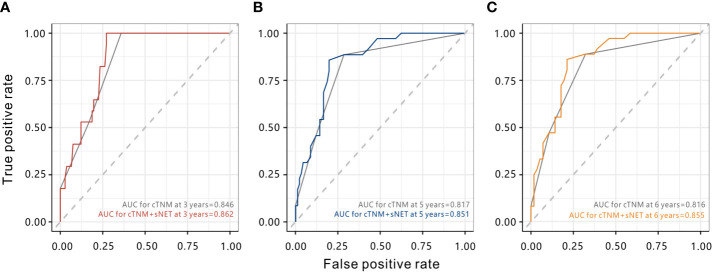
Time-dependent ROC curves to compare the prognostic accuracy of the combination of stromal NETs density and the clinical TNM stage with the clinical TNM stage alone. **(A)** AUCs at 3 years. **(B)** AUCs at 5 years. **(C)** AUCs at 6 years. cTMN, clinical TNM stage. sNET, stromal NET density.

To improve the prognostic accuracy of the current TNM staging system, we established a predictive model for cervical cancer by combining the TNM staging system and stromal NET density using tROC. The combination of both factors achieved higher AUC value than the TNM staging system (0.862 *vs.* 0.846 at 3 years, *P* = 0.172; 0.851 *vs.* 0.817 at 5 years, *P* = 0.010; 0.855 *vs.* 0.816 at 6 years, *P* = 0.023), with significant differences at five years and six years (**Figure 6**). The AUC value combining the TNM staging system and stromal CD66b^+^ neutrophil density had no statistical difference (data not shown). The results suggested that combining stromal NET density and the TNM staging system had better prognostic accuracy for cervical cancer than TNM staging alone.

## Discussion

This is the first study to show that PMNs in cervical cancer are activated to produce NETs and thus suggests a possible role for NETs in cervical cancer pathogenesis. We employed quantitative MIF to enable objective and compartment-specific measurement of PMNs and NETs in the cervical cancer microenvironment for outcome prediction. We found that higher densities of stromal PMNs and NETs were correlated with poor outcome in a retrospective cohort of patients with cervical cancer, and high stromal NET density was an independent prognostic factor for RFS. Combining NET status and the TNM staging system yielded better prognostic accuracy for cervical cancer than TNM staging system alone. Thus, patients with high stromal PMNs and NETs densities should be carefully followed. Stromal NET density may also serve as a new stratification factor for randomized trials. These results suggest that targeting NETs may be an approach to prevent tumor progression, but they require validation in an independent and larger population.

Previous studies demonstrate that the tumor microenvironment plays a crucial role in cancer formation, progression and metastasis (5, 32, 33). Cancer-related inflammation has emerged as the seventh hallmark of the disease (34). Within the tumor microenvironment, inflammatory cells—including neutrophils—influence almost every aspect of cancer including initiation, angiogenesis, invasion, and dissemination (35). Neutrophils make up a significant portion of the inflammatory cell infiltrate findings in a wide variety of human cancers and murine models (36). There is increasing evidence indicating that neutrophils play vital functions in the tumor microenvironment (37). Some pro-inflammatory factors in the tumor microenvironment have been reported to extend neutrophil survival time, such as the interferon-γ, granulocyte colony-stimulating factor (G-CSF) or transforming growth factor-β (10, 37, 38). These cytokines activate tumor-associated neutrophils in different conditions, which results in anti- and pro-tumor functions of neutrophils (39).

Although neutrophils have the potential to suppress tumor growth and damage the vascular endothelium through oxidants and proteolytic enzymes (40), tumor-associated neutrophils are generally considered a pro-tumor factor in multiple cancers (10, 41, 42). Using over 5000 cases of 25 different cancer types, Gentles et al. indicated that higher infiltration of PMNs (including neutrophils) was associated with the lowest overall survival rate compared to other leukocytes (43). Another study of neutrophils reported that they could establish a pre-metastatic niche for the malignant tumor cells (44). We previously demonstrated that cytokines like G-CSF and interleukin (IL)-6 in the tumor environment could modulate neutrophil function and enable them to promote tumor growth and angiogenesis (45).

The CD66b^+^ phenotype is unique to neutrophils and may reflect a specific activated subtype of (46). Carus *et al.* reported that elevated CD66b^+^ tumor associated PMN count was an independent prognostic factor for recurrence in localized (stages Ib and IIa) cervical cancer (47). In this study, we extended this observation to stage I-IV cervical cancer, further clarifying the impact of neutrophils on patient outcome. We observed that neutrophils were present predominantly in the stromal tissue. Specifically, a high stromal CD66b^+^ neutrophil density instead of tumor nest CD66b^+^ neutrophil density was significantly associated with short RFS. Low stromal CD66b^+^ neutrophil density correlated with favorable RFS in univariate analysis but was not an independent prognostic factor in multivariate analysis.

Following activation by stimuli such as PMA or lipopolysaccharide (LPS) or an infectious environment, neutrophils produce the fiber-like net by ejecting nuclear chromatin attached to proteases (e.g., NE, matrix metalloproteinase-9, MPO) to entangle and eliminate the substance or pathogen (48). Neutrophils are also activated and form NETs in the tumor microenvironment. NETs play a pro-tumor role during tumor progression and metastasis by enhancing angiogenesis, extracellular matrix remodeling, and tumor cell proliferation through proteases such as MPO or NE (49) or by trapping the circulating cancer cells and promoting metastasis and disease recurrence (50). Compared to healthy controls, NET levels were higher in cancer patient plasma and tissues such as lung cancer, pancreatic adenocarcinoma, bladder cancer, gastric cancer and cervical cancer (18, 51, 52). NETs were also detected in tumor tissues and metastatic lymph nodes from 10 patients with colon cancer (53). Furthermore, among eight patients with Ewing sarcoma, Tumor-associated NETs were discovered in patients who had a poor prognosis (54). Adverse patient outcomes are associated with increased preoperative NET production in patients with Colorectal Cancer (55). High NET levels in serum and tumor tissue are associated with poor prognosis in patients with diffuse large B cell lymphoma (DLBCL). DLBCL-derived IL-8 interacted with its receptor on neutrophils to form NETs, resulting in upregulation of the Toll-like receptor 9 and its downstream signaling pathway to promote tumor progression (25). In breast cancer, LPS-activated neutrophils induced NET formation and converted dormant cancer cells to aggressive lung metastases (56).

A previous study demonstrated that peripheral blood neutrophils generate NETs in 53.57% of cervical cancer patients before treatment, but there are no NETs in healthy people (18). However, it remains unclear whether NETs play a role in cervical cancer progression. Here, we provide evidence that enhanced NET formation was associated with poor survival in cervical cancer patients, indicating that NETs can be a potential prognostic marker and therapeutic target for this disease. Additional mechanisms of NET function in the tumor environment need to be investigated in the future.

We found that NET number but not PMN number was an independent prognostic factor for RFS in cervical cancer patients. Two possible reasons are: (i) there are different tumor environment factors in early and late stage of cervical cancer that could modulate neutrophil function and allow them to have pro- or anti-tumor functions and (ii) NETs are reflective of PMN activity in the tumor environment and may better reflect the pro-tumor functional status of PMNs, supporting the hypothesis that the neutrophils exert pro-tumor effects through multiple mechanisms.

The TNM staging system is considered the gold standard for clinical staging a variety of malignancies; however, this system is based almost exclusively on the anatomical spread of cancer and only narrowly examines the tumor cells without considering the host reaction (3, 56). The intratumoral inflammatory reaction is an important parameter influencing the natural course of the disease. Diverse immune cell types including CD20+ B cells, CD3+ T cells, CD56+ natural killer (NK) cells, and CD68+ macrophages and immune markers PD-L1 were detected in different proportions of cervical carcinoma (57), and these cellular and molecular indicators are typically associated with patient’ survival (58). In this retrospective study, the combination of NET density and TNM staging as a prognostic model is more predictive than using TNM staging alone. NETs, which may reflect the status of the tumor microenvironment of patients as a molecular indicator, may be used as a supplement to TNM staging or may be a candidate indicator for molecular classification of cervical cancer, and may become a potential therapeutic target. In the future, we will expand the sample size, design prospective studies and include more immune-indicators to further verify the above results.

A major strength of our study is the systematic assessment of PMNs and NETs in the different compartments (i.e., tumor nest and stromal tissue) with an objective and quantitative MIF method, and their correlations with RFS. We confirmed the findings of the highest densities of CD66b^+^ neutrophils in the stromal compartment and demonstrated an independent prognostic capacity for NETs. This may reflect the importance of NET activity in tumor border migration and should be further studied.

The study has some shortcomings. It was retrospective and performed at a single center. There was also a lack of some clinical prognostic factors due to the use of TNM staging instead of FIGO staging. The study was also limited by its small sample size, which may explain the lack of statistical significance in clinical stage subgroup analysis of high- and low-density NETs. Although we found that higher levels of PMN and NET infiltration were associated with tumor progression, further studies are needed to clarify the underlying mechanism.

In conclusion, this study provides the first evidence that PMNs release NETs in cervical cancer tumor tissue. Increased NET density is an independent prognostic factor for short RFS in patients with cervical cancer. Combining assessments of clinical stage with NET assessment may further improve prognostic stratification. These results could lead to the development of new therapeutic strategies for cervical cancer.

## Data Availability Statement

The original contributions presented in the study are included in the article/**Supplementary Material**. Further inquiries can be directed to the corresponding authors.

## Ethics Statement 

The studies involving human participants were reviewed and approved by Ethics Committee of Hubei Maternal and Child Health hospital. The patients/participants provided their written informed consent to participate in this study.

## Author Contributions

BY, XW, and QM conceived and designed the study. BY conducted the experiments and wrote the manuscript. BY, XW, QM, and XD collected the data. BY and XD performed the data analysis and data interpretation. XW and QM supervised all aspects of the studies. All authors contributed to the article and approved the submitted version.

## Funding

The present study was supported by the National Natural Science Foundation of China (grant no. 81502265), Innovation Team Program of Science and Technology Department of Hubei Province (grant no. 2017CKC891), General Program of Health and Safety Commission of Hubei Province (grant no. WJ2018H0144, WJ2019M228).

## Conflict of Interest

The authors declare that the research was conducted in the absence of any commercial or financial relationships that could be construed as a potential conflict of interest.

## Publisher’s Note

All claims expressed in this article are solely those of the authors and do not necessarily represent those of their affiliated organizations, or those of the publisher, the editors and the reviewers. Any product that may be evaluated in this article, or claim that may be made by its manufacturer, is not guaranteed or endorsed by the publisher.
