# Supplementary material for: Stromal Neutrophil Extracellular Trap Density Is an Independent Prognostic Factor for Cervical Cancer Recurrence
Source: Front Oncol. 2021 Aug 11;11:659445. doi: 10.3389/fonc.2021.659445 (PMC8386589; doi:10.3389/fonc.2021.659445)
Supplement: Supplementary file 1 [file Table_1.docx]

# Supplementary Material

Supplementary Table 1. Primary antibodies and opal fluorophores

| **Antibodies** | **Dilution** | **Clone, source** | **Company** | **Catalog Number** | **Opal fluorophore** |
| --- | --- | --- | --- | --- | --- |
| Anti-CK | 1:5 | Mouse/Rabbit polyclonal | MXBiotechnology | kit-0009 | 520 |
| Anti-CD66b | 1:500 | Mouse monoclonal | Arigobio | ARG65820 | 570 |
| Anti-MPO | 1:1000 | Mouse monoclonal | Proteintech | 66177-1-Ig | 650 |
| Anti-Histone H3Cit | 1:3000 | Rabbit polyclonal | Abcam | Ab5103 | 620 |

CK, cytokeratin; MPO, myeloperoxidase
